# Family Mealtimes in Hispanic Households: Associations of General Parenting Styles and Food Parenting Practices with Adolescent Food Group Intake

**DOI:** 10.3390/nu18193129

**Published:** 2026-09-23

**Authors:** Lillie Monroe-Lord, Azam Ardakani, Elmira Asongwed, Dorothy Wakefield, Tia Jeffery, Phronie Jackson, Eyerusalem Beza

**Affiliations:** 1 College of Agriculture, Urban Sustainability and Environmental Sciences, University of the District of Columbia, Washington, DC 20008, USA; azam.ardakani@udc.edu (A.A.);; 2 Independent Statistical Consultant, Storrs, CT 06268, USA

**Keywords:** family meals, Hispanic adolescents, parenting styles, food parenting practices, dietary intake

## Abstract

*Background/Objectives*: Hispanic adolescents experience disproportionately high rates of obesity and diet-related health risks, yet the family and parenting factors that shape their dietary behaviors remain incompletely understood. This study examined associations between parent sociodemographic characteristics and family meal frequency; among general parenting styles, food parenting practices, and family meal frequency; and between family meal frequency and adolescent intake across major USDA food groups, among Hispanic parent–adolescent dyads. *Methods*: A cross-sectional online survey was conducted among 251 Hispanic parent–adolescent dyads (adolescents aged 10–17 years). Parents reported sociodemographic characteristics, general parenting styles (Comprehensive General Parenting Questionnaire), and food parenting practices (Comprehensive Feeding Practices Questionnaire). Adolescents self-reported dietary intake using the Youth/Adolescent Food Frequency Questionnaire. Family meal frequency was categorized as ≤2, 3–6, or ≥7 meals per week. *Results*: Older parents (*p* = 0.034) and married parents (*p* = 0.036) reported more frequent family meals than younger and unmarried parents. In adjusted multivariable models, more frequent family meals were associated with greater parental responsiveness (overall *p* = 0.009), involvement (overall *p* = 0.006), expectations (overall *p* = 0.047), monitoring (overall *p* = 0.037), and encouragement (overall *p* < 0.001). None of the five food group outcomes—dairy, fruit, grains, protein foods, or vegetables—showed a significant overall adjusted association with family meal frequency. *Conclusions*: Frequent family meals appear closely connected with supportive parenting practices among Hispanic adolescents, independent of sociodemographic characteristics, but were not independently associated with intake of any specific food group. Interventions to improve adolescent nutrition in Hispanic families may benefit from promoting both regular family meals and supportive food parenting practices.

## 1. Introduction

Adolescence is a critical period for nutritional intervention, characterized by rapid physical growth, hormonal changes, and increasing autonomy in dietary choices [1]. Diet quality commonly declines during this period as adolescents consume fewer nutrient-rich foods and more energy-dense, nutrient-poor foods [1,2]. Many U.S. adolescents do not meet the Dietary Guidelines for Americans: the national average Healthy Eating Index (HEI-2020) score among U.S. children and adolescents aged 2–18 is 54 out of 100 and declines from approximately 61 among children aged 2–5 years to 48–51 among adolescents aged 12–18 years [3]. These concerns are particularly pronounced for Hispanic adolescents, whose obesity prevalence is higher than that of non-Hispanic White adolescents (approximately 26.2% vs. 16.6%) [4] and who experience elevated risk insulin resistance, elevated triglycerides, and type 2 diabetes [5]. Identifying modifiable family influences on diet is therefore a public health priority. 

A family meal is an occasion when most or all immediate household members eat together [6], often providing the caregiver prepared food and an opportunity for conversation [7]. Frequent family meals have been associated with healthier dietary patterns among children and adolescents [8,9,10,11,12], including greater fruit and vegetable intake and more favorable weight-related outcomes [13]. Family meals during adolescence have also predicted higher fruit, vegetable, and calcium-rich food intake and lower soft-drink consumption in young adulthood [10]. An umbrella review further identified regular family meals as protective for diet quality, obesity risk, health-risk behaviors, and mental health [11]. Shared meals have particular cultural significance in Hispanic and Latino families [14,15,16,17,18,19]. Qualitative studies indicate that many families prioritize eating together despite work schedules, childcare responsibilities, and other household demands [14,15,16]. Latina mothers frequently assume primary responsibility for meal planning and preparation and may reserve family meals for weekends because of weekday constraints [18]. Nevertheless, national data indicate that daily family meals decline from more than 60% among Hispanic preschoolers to 38% among Hispanic adolescents aged 13–17 years [19]. 

Family meals occur within a broader parenting context. General parenting style describes the general emotional climate of caregiving, whereas food parenting practices are specific strategies used to influence children’s eating [20]. Building on Baumrind’s model [21], later expanded by Maccoby and Martin [22], general parenting styles are generally classified as authoritative, authoritarian, permissive (indulgent), and neglectful (uninvolved), defined along the following two dimensions: warmth/responsiveness and control/demandingness. Food parenting practices, in turn, are classified into the following three domains: coercive control (e.g., pressure to eat, restriction, and use of food as reward), structure (e.g., meal routines, food availability, and parental modeling), and autonomy support (encouraging independent food choices and self-regulation) [20]. These parenting approaches often manifest directly during family meals: authoritative parents, for example, are more likely to maintain regular and structured mealtime routines [23]. Among Hispanic families, authoritative mothers are also more likely to use supportive rather than controlling feeding practices [24], while greater maternal acculturation has been associated with more positive, autonomy-supportive practices [25]. 

Family meal frequency also varies across sociodemographic groups. Fewer than half of adolescents report frequent family meals, with higher prevalence among boys than girls [26]. Household structure is influential: mothers in two-parent households have higher odds than fathers of eating at home with their children and participating in family dinners, while among single-parent households, men have lower odds of family meals than partnered or married men [27]. Findings on socioeconomic status are mixed: cross-national data show higher-socioeconomic-status households share daily family meals more often [28], while U.S. NHANES data show more frequent family meals among lower-income households and those headed by older or married adults [29]. Parental education appears more consistently and positively associated with family meal frequency: adolescents with a college-educated parent are 36% more likely to report frequent family meals than those without [26]. However, these relationships remain understudied among Hispanic families. Associations may also differ across food groups. Family meals are consistently associated with greater fruit and vegetable consumption [12,30]. Among Hispanic preschoolers, however, whole-grain intake was the only dietary component associated with shared meal frequency [31]. Polish adolescents who ate daily family meals were also more likely to consume dairy products regularly [32]. Evidence regarding grains, dairy, and protein foods among U.S. Hispanic adolescents remains limited. Although family meals have been associated with healthier adolescent diets, few studies have examined these relationships in Hispanic populations or simultaneously evaluated general parenting styles alongside multiple food parenting practices; it also remains unclear which food groups are most sensitive to family meal frequency, and the role of sociodemographic factors in shaping mealtime routines and parenting behaviors concurrently has rarely been examined. The present study addresses these gaps with the following three specific objectives: (1) to examine associations between parent sociodemographic and weight-related characteristics and family meal frequency; (2) to examine associations between general parenting styles, food parenting practices, and family meal frequency; and (3) to examine associations between family meal frequency and adolescent intake across major USDA food groups (fruits, vegetables, grains, dairy, and protein foods), among Hispanic parent–adolescent dyads. Together, these objectives aim to clarify how mealtime routines and parenting behaviors relate to adolescent dietary patterns in a population at elevated nutritional risk.

## 2. Materials and Methods

### 2.1. Study Design and Participants

This cross-sectional study was conducted between November and December 2021 and included 251 Hispanic parent–adolescent dyads. Participants were recruited through Qualtrics, an online survey panel provider. The sampling frame consisted of members of Qualtrics-affiliated U.S. online research panels, and a nonprobability panel-sampling approach was used. Potentially eligible adult panel members were invited based on their panel profiles and completed screening questions to confirm eligibility. An a priori power analysis conducted before recruitment indicated that a minimum of 200 parent–adolescent dyads were required for the planned analyses. The study was approved by the Institutional Review Board (IRB) of the University of the District of Columbia. Eligible respondents were parents or primary caregivers who self-identified as Hispanic or Latino, were responsible for feeding an adolescent aged 10–17 years, were literate in English, had internet access, and were willing to participate with their adolescent. Parents provided informed consent, and adolescents provided assent prior to completing the survey. To enhance data quality, Qualtrics applied standard panel-level screening procedures, including IP-address screening, to reduce duplicate or potentially fraudulent responses. Survey responses were additionally reviewed for eligibility, completeness, and consistency of key demographic information before inclusion in the analytic sample.

The study was conducted in accordance with the Declaration of Helsinki and approved by the Institutional Review Board (or Ethics Committee) of the University of the District of Columbia (protocol code IRB#878591-2, 16 February 2021).

### 2.2. Demographic Characteristics

Demographic information was collected from both parents and adolescents. Parents reported their own age, sex, race/ethnicity, educational attainment, marital and employment status, household income, household composition, primary language spoken at home, and participation in food assistance programs, as well as their adolescent’s age, sex, race/ethnicity, and state of residence. Adolescents independently reported their own age, sex, and race/ethnicity in a separate section to enable cross-validation.

### 2.3. General Parenting Styles and Food Parenting Practices

General parenting styles and food parenting practices were assessed using established self-report instruments, both completed by the parents regarding their own parenting behaviors. General parenting style was measured using the following seven subscales of the Comprehensive General Parenting Questionnaire (CGPQ) [33,34]: responsiveness, involvement, inconsistent discipline, expectations, psychological control, authoritarian behaviors, and excessive involvement. These subscales were selected a priori because they capture both supportive parenting dimensions (warmth, sensitivity, and active engagement) and less supportive dimensions (inconsistent rule enforcement, psychological control, and intrusive involvement) most directly relevant to mealtime interactions, while limiting respondent burden; this subset has precedent in prior applications of the CGPQ in feeding-focused research [34].

Food parenting practices were assessed using the following four subscales of the Comprehensive Feeding Practices Questionnaire (CFPQ) [35]: restriction, modeling, monitoring, and encouragement. These subscales were selected because they represent the food parenting constructs most consistently linked to child dietary intake and weight outcomes in prior validation and applied research [20,35], spanning both structure-based (modeling, monitoring) and coercive-control (restriction) domains, alongside a supportive, autonomy-oriented practice (encouragement). Monitoring assessed the extent to which parents kept track of their adolescent’s intake of foods such as sweets, sugary drinks, snack foods, and high-fat foods. Encouragement assessed parents’ use of praise, support, and reasoning to promote healthier eating. Modeling captured the extent to which parents demonstrated healthy eating behaviors themselves. Restriction reflected parental efforts to limit adolescents’ access to less healthy foods for health- or weight-related reasons.

### 2.4. Food Group Intake

Adolescent dietary intake was self-reported using the 2012 Youth/Adolescent Food Frequency Questionnaire (FFQ), which captures the type, frequency, and portion size of foods consumed over the past year [36,37]. Following standard FFQ scoring procedures [37], reported frequencies and portion sizes for individual food items were converted to weekly servings and summed within each of the following five major food groups: fruits, vegetables, grains, dairy, and protein foods, using the food group classifications specified in the Dietary Guidelines for Americans [38]. These food groups were selected because they represent the core components of the Dietary Guidelines for Americans and are central to recommendations for healthy dietary patterns.

### 2.5. Weight Status Assessment

Weight status was based on self-reported height and weight, converted to metric units to calculate BMI (kg/m^2^). Parent BMI was classified using standard adult cutoffs (normal, 18.5–24.9; overweight, 25.0–29.9; and obese, ≥30.0 kg/m^2^) [39]. Adolescent BMI-for-age percentiles were derived from CDC 2000 sex- and age-specific reference data [40] and classified as healthy weight (5th–<85th percentile, including the small underweight subgroup), overweight (85th–<95th), or obese (≥95th) [41]. Implausible values were flagged and set to missing per standard CDC range-check procedures, and participants missing valid height/weight were excluded from weight-status analyses.

### 2.6. Family Meal Frequency

Family meal frequency was assessed via parent report using the question: “During the past 7 days, how many times did all, or most, of your family in your house eat a meal together?” Participants selected from six response options ranging from “Never” to “More than 7 times.” For analysis, responses were grouped into the following three categories: ≤2, 3–6, and ≥7 times/week.

### 2.7. Statistical Analysis

Descriptive statistics were used to summarize demographic, socioeconomic, health, and behavioral characteristics. Categorical variables are reported as frequencies and percentages, and continuous variables as means with standard deviations or medians with interquartile ranges, as appropriate. In line with Objective 1, associations between parent sociodemographic and weight-related characteristics and family meal frequency were evaluated using chi-square tests. In line with Objectives 2 and 3, differences in weekly intake of major food groups (fruits, vegetables, grains, dairy, and protein foods) and in parenting constructs (general parenting styles and food parenting practices) across family meal frequency categories were assessed using the Kruskal–Wallis test. Nonparametric tests were selected given the categorical/ordinal nature of family meal frequency and the skewed distributions of the parenting and dietary intake variables. 

For the adjusted analyses, 16 separate multivariable linear regression models were fitted: one for each of the 11 parenting constructs and five food group outcomes. Family meal frequency was entered as a categorical variable, with ≤2 meals/week as the reference category. Models were adjusted for adolescent age and sex, parent age group and sex, parental education, household income, and marital status. HC3 heteroskedasticity-robust standard errors were used. Unstandardized regression coefficients (β) and 95% confidence intervals are reported; the overall association of family meal frequency with each outcome was evaluated using a two-degree-of-freedom Wald test. Three dyads with missing household income were excluded by complete-case analysis, giving an adjusted sample of 248. Parent and adolescent weight status were not included in the primary adjusted models; they remained in the descriptive and bivariate analyses. Statistical significance was defined as *p* < 0.05. The descriptive and bivariate analyses were conducted using SAS software (version 9.4, SAS Institute Inc., Cary, NC, USA).

## 3. Results

Table 1 summarizes the demographic, sociodemographic, health, and behavioral characteristics of Hispanic adolescents and their primary caregivers, including family meal frequency. The sample included 251 adolescents aged 10–17 years (mean age = 13.94, SD = 2.17); 57.0% were aged 14–17 years, and 53.4% were female. Most adolescents had normal weight status (56.9%), while 20.2% were overweight and 22.9% were obese. Among caregivers, 65.7% were aged ≥35 years and 64.9% were female. Mean parental body mass index was 26.98 (SD = 6.13), with 42.3% classified as normal weight, 32.1% as overweight, and 25.6% as obese.

Over two-thirds (69.3%) of parents had at least some college education, 60.1% reported annual household incomes above $45,000, 65.7% were married, and 93.2% were a parent, including a step or a foster parent. Regarding family mealtime behavior, 21.9% reported eating together ≤2 times/week, 45.4% reported 3–6 times/week, and 32.7% ate together ≥7 times/week.

Addressing Objective 1, sociodemographic and weight-related characteristics were examined in relation to family meal frequency. Significant associations were observed for parent age (*p* = 0.034) and marital status (*p* = 0.036). Parents aged ≥35 years were more likely than younger parents to report family meals ≥7 times/week (37.6% vs. 23.3%), and married parents were more likely than unmarried parents to report this level of meal frequency (38.2% vs. 22.1%). Although not statistically significant, parents with more than a high school education were more likely to report family meals 3–6 times/week than those with a high school education or less (50.0% vs. 35.1%; *p* = 0.069). Household income was not significantly associated with family meal frequency (*p* = 0.177). Family meal frequency was not significantly associated with adolescent age (*p* = 0.431), adolescent sex (*p* = 0.099), parent sex (*p* = 0.130), parent weight status (*p* = 0.798), or adolescent weight status (*p* = 0.770) (Table 2).

Addressing Objective 3, the Kruskal–Wallis test was used to examine differences in weekly servings of major food groups across family meal frequency categories. Of the food groups assessed, only grain servings differed significantly across categories, χ^2^(2) = 7.77, *p* = 0.020.

Analysis indicated that adolescents who shared family meals 3–6 times or ≥7 times/week had higher median grain intake than those who shared family meals ≤2 times/week. No significant differences were observed for dairy (*p* = 0.367), fruit (*p* = 0.386), protein (*p* = 0.205), or vegetable (*p* = 0.637) intake (Table 3).

Addressing Objective 2, the Kruskal–Wallis test was used to examine differences in general parenting styles and food parenting practices across family meal frequency categories (Table 4; Figure 1). Significant differences were observed for several parenting constructs. Median parental responsiveness differed across groups, χ^2^(2) = 15.35, *p* < 0.001, with higher scores among adolescents reporting ≥7 shared meals per week. Similar patterns were observed for parental involvement, χ^2^(2) = 12.59, *p* = 0.002, and parental expectations, χ^2^(2) = 8.30, *p* = 0.016.

Significant differences were also observed for food parenting practices. Median monitoring scores varied across family meal frequency categories, χ^2^(2) = 12.83, *p* = 0.002, and encouragement showed the strongest association with meal frequency, χ^2^(2) = 22.91, *p* < 0.001, with higher scores among families reporting more frequent shared meals. No significant differences were detected for inconsistent discipline, psychological control, authoritarian parenting, excessive involvement, restriction, or modeling (all *p* > 0.05) (Table 4; Figure 1).

In adjusted multivariable models (Table 5), several of the associations between family meal frequency and parenting constructs observed in bivariate analysis remained significant after adjustment for sociodemographic covariates. Compared with adolescents whose families shared ≤2 meals/week, those in families with ≥7 meals/week had significantly higher adjusted scores for parental responsiveness (β = 0.37, 95% CI: 0.10, 0.65; *p* = 0.008; and overall *p* = 0.009), involvement (β = 0.44, 95% CI: 0.15, 0.73; *p* = 0.003; and overall *p* = 0.006), expectations (β = 0.38, 95% CI: 0.07, 0.68; *p* = 0.015; and overall *p* = 0.047), monitoring (β = 0.49, 95% CI: 0.10, 0.87; *p* = 0.013; and overall *p* = 0.037), and encouragement (β = 0.55, 95% CI: 0.22, 0.88; *p* = 0.001; and overall *p* < 0.001). No significant adjusted associations were observed for inconsistent discipline, psychological control, authoritarian parenting, excessive involvement, restriction, or modeling (all overall *p* > 0.05).

In adjusted models for adolescent food group intake (Table 6), none of the following five weekly food group outcomes showed a statistically significant overall adjusted association with family meal frequency: dairy (overall *p* = 0.402), fruit (overall *p* = 0.080), grains (overall *p* = 0.101), protein foods (overall *p* = 0.370), or vegetables (overall *p* = 0.410). The bivariate association between family meal frequency and grain intake (Table 3) attenuated after adjustment for sociodemographic covariates and was no longer statistically significant overall, although the ≥7 vs. ≤2-meals/week contrast remained nominally significant (β = 8.19, 95% CI: 0.65, 15.72; and *p* = 0.033). Similarly, the ≥7 vs. ≤2 meals/week contrast for fruit intake was nominally significant (β = 6.59, 95% CI: 0.78, 12.41; and *p* = 0.026), but the overall 2-degree-of-freedom test for fruit was not significant, so neither pairwise result was interpreted as evidence of an association. No pairwise contrasts for dairy, protein, or vegetable intake were significant.

## 4. Discussion

This study examined how sociodemographic characteristics, general parenting styles, and food parenting practices relate to family meal frequency, and how family meal frequency in turn relates to adolescent food group intake, among Hispanic parent–adolescent dyads.

Consistent with our first objective, family meal frequency was significantly associated with parent age and marital status. Parents aged ≥35 years were more likely to share meals with their adolescents ≥7 times/week than younger parents. Despite limited prior evidence linking parental age to family meal frequency in Hispanic populations specifically, this finding may reflect greater routine stability and prioritization of family time among older caregivers, representing a novel contribution to the literature. Marital status has been more widely studied: consistent with our findings, Larson et al. [42] reported that adolescents in two-parent households, including Hispanic youth, participated in family meals more frequently than those in single-parent households, and Lebron et al. [43] found that mealtime communication was more positive among adolescents in dual-parent households. These findings highlight family structure as an important context for shared mealtime routines among Hispanic youth.

Although parental education and household income were not significantly associated with family meal frequency in this study, these socioeconomic factors may affect nutrition knowledge, food affordability, work schedules, and opportunities for shared meals [26,28,29]. The HELENA study similarly found that higher parental educational level was associated with better adolescent diet quality in northern, but not southern, Europe, highlighting the potential importance of cultural and geographic context [44]. Cultural values may support shared meals in Hispanic families, whereas acculturation may modify meal routines, dietary preferences, and food parenting practices [14,15,16,17,18,19,25]. Because acculturation was not examined in the present analysis, its potential contribution should be evaluated in future studies.

Our bivariate finding that grain intake was the only food group significantly associated with family meal frequency echoed Power et al. [31], who reported a similar pattern among Hispanic preschoolers. In our study, however, the association was attenuated after adjustment, suggesting that sociodemographic differences may partly explain the finding. Differences from prior work may also reflect participants’ ages, dietary assessment methods, and our inability to distinguish whole from refined grains. Although family meals have been associated with better overall diet quality [45], meal frequency alone may not adequately capture meal composition or quality. Future studies should examine these relationships using longitudinal designs and more detailed dietary measures.

More frequent family meals were associated with higher levels of parental responsiveness, involvement, expectations, monitoring, and encouragement, a pattern of warmer, more supportive, and more structured parenting, without greater reliance on controlling behaviors. This aligns with prior research identifying general parenting style and home environment as key correlates of family meal frequency and diet quality [31,46,47,48,49]. Berge et al. [23] similarly reported that authoritative parenting was positively associated with more frequent family meals among adolescents over time, and Power et al. [31] found that family meals and food parenting practices, particularly monitoring and encouragement, were jointly associated with healthier dietary behaviors among preschoolers. Monitoring in particular has been linked to greater adherence to dietary recommendations among children [46]. Notably, family meal frequency was not associated with restriction or other controlling parenting dimensions: modeling also showed no association. This pattern suggests that frequent family meals co-occur with supportive structure rather than coercive practices. Given the limited research examining these relationships across multiple parenting domains in Hispanic families specifically, our findings extend the literature by showing that frequent family meals are associated not only with shared eating occasions but with a broader pattern of supportive parenting characteristics.

This study has several strengths, including its focus on Hispanic parent–adolescent dyads, an understudied population at elevated risk for diet-related health disparities; the use of validated measures of general parenting styles and food parenting practices; and assessment of all major food groups, enabling a comprehensive view of how family meals relate to both parenting behaviors and adolescent dietary intake.

Several limitations should be considered. Because the parenting constructs and family meal frequency were reported by the same parent, shared-method effects may account for some associations. Dietary intake was independently reported by adolescents, reducing—but not eliminating—this concern for the food group analyses. The sample was also not characterized by Hispanic subgroup heritage, generational status, acculturation, or region, limiting generalizability to the broader U.S. Hispanic population, and recruitment through an online panel meant parental education was higher than in many comparison studies, limiting direct comparability. Additional limitations include the cross-sectional design, which prevents establishing temporal ordering or causality; therefore, the findings should be interpreted as associations rather than causal effects; reliance on self-reported data, which introduces recall and social desirability bias; a single-item, parent-reported measure of family meal frequency that did not capture meal type, duration, or quality; data collection during the COVID-19 pandemic, which may have affected typical mealtime routines; the lower reliability of food frequency measures relative to methods such as 24 h recalls; and unassessed contextual factors such as food insecurity and parental work schedules.

## 5. Conclusions

Overall, this study found that more frequent family meals among Hispanic parent–adolescent dyads were associated with greater parental responsiveness, involvement, expectations, monitoring, and encouragement after adjustment for sociodemographic factors; however, no individual food group showed a significant overall adjusted association with family meal frequency. Older and married parents were also more likely to report regular family meals in the bivariate analyses. These findings suggest that frequent shared meals may co-occur with a broader supportive home environment. However, the cross-sectional design prevents establishing temporal direction or causality. Future longitudinal and intervention studies are needed to confirm these associations, evaluate meal quality, incorporate multiple reporters, and account for broader contextual factors such as acculturation, food insecurity, Hispanic subgroup heritage, and parental work schedules.

## Figures and Tables

**Figure 1:**
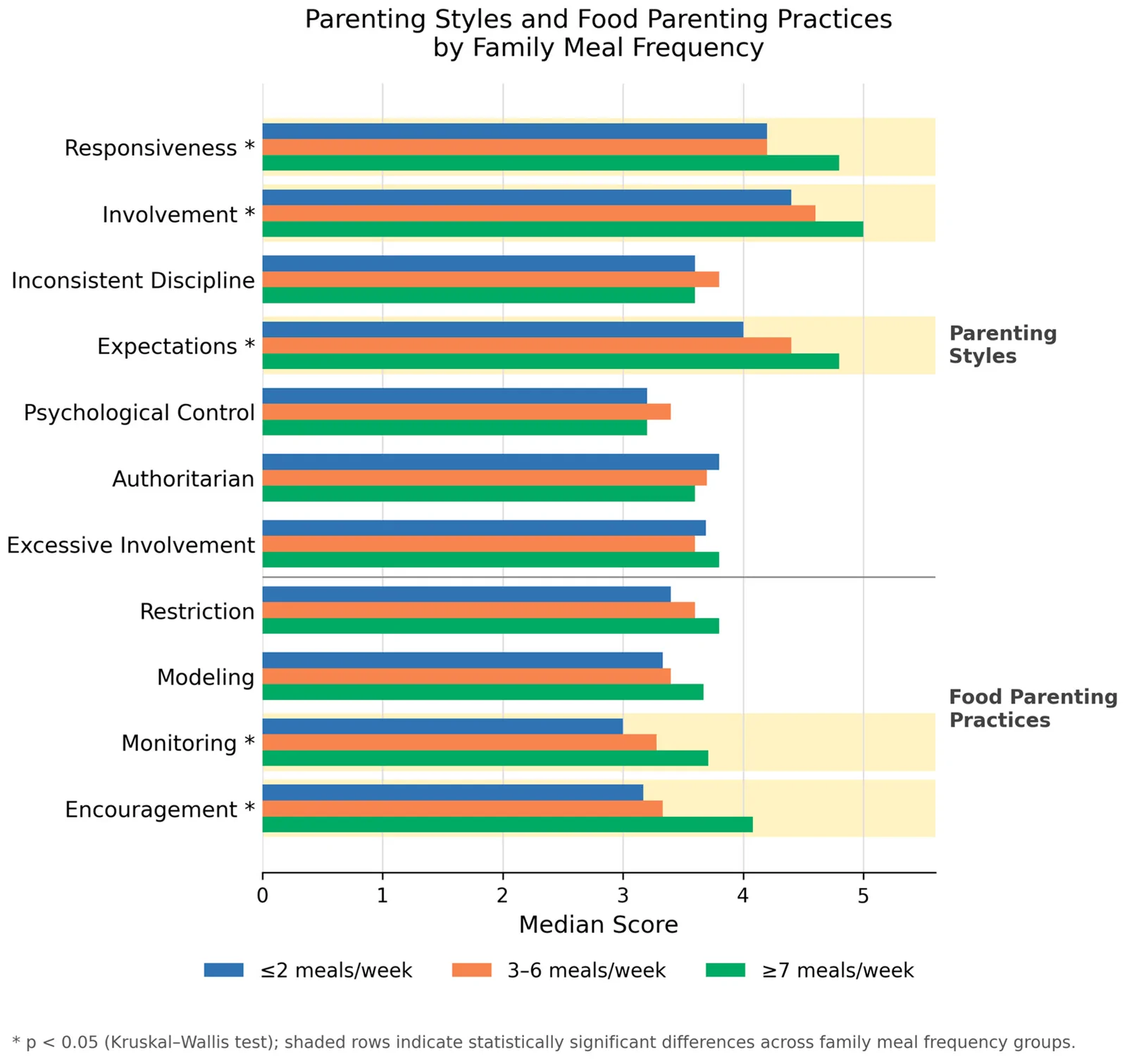
General parenting styles and food parenting practices by family meal frequency. Note: Shaded rows indicate statistically significant differences across family meal frequency groups (Kruskal–Wallis test, *p* < 0.05). Bars represent median scores.

**Table 1:** Demographic, sociodemographic, health, and behavioral characteristics of Hispanic adolescents and their caregivers.

Characteristics	N (%)	Mean (SD)	Median
Demographic Characteristics			
Adolescent age (years)		13.94 (2.17)	
10–13	108 (43.03)		
14–17	143 (56.97)		
Adolescent sex			
Female	134 (53.39)		
Male	117 (46.61)		
Parent age			
<35	86 (34.26)		
≥35	165 (65.74)		
Parent sex			
Female	163 (64.94)		
Male	88 (35.06)		
Health Characteristics			
Adolescent’s weight status		69.85 (28.95)	69.85
Normal	124 (56.88)		
Overweight	44 (20.18)		
Obese	50 (22.94)		
Missing	33		
Parents’ weight status		26.98 (6.13)	26.98
Normal	91 (42.33)		
Overweight	69 (32.09)		
Obese	55 (25.58)		
Missing	36		
Socioeconomic Characteristics			
Parents’ education			
Below HS	17 (6.77)		
Diploma or GED	60 (23.90)		
Some college/technical school	67 (26.69)		
≥4 years of college	107 (42.63)		
Annual household income (USD)			
Less than $25,000	35 (13.94)		
$25,000–$44,999	64 (25.50)		
$45,000–$64,999	52 (20.72)		
$65,000–$84,999	24 (9.56)		
More than $85,000	73 (29.08)		
Marital status			
Not married	86 (34.26)		
Married	165 (65.74)		
Relationship with adolescent			
Parent (incl. step or foster parent)	234 (93.23)		
Aunt/uncle	5 (1.99)		
Grandparent	3 (1.20)		
Sibling	9 (3.59)		
Behavioral Characteristics			
Meals together			
2 or fewer times	55 (21.91)		
3–6 times	114 (45.42)		
7 or more times	82 (32.67)		

Note: Percentages reflect valid (non-missing) responses. HS: high school diploma. GED: General Educational Development credential, an alternative high-school-equivalency credential.

**Table 2:** Sociodemographic and weight status characteristics by family meal frequency among Hispanic families.

	2 or Fewer	3–6 Times	7 or More	χ^2^ (*df*)	*p*-Value
Adolescent age				1.68 (2)	0.431
10–13	20 (18.52)	49 (45.37)	39 (36.11)		
14–17	35 (24.48)	65 (45.45)	43 (30.07)		
Adolescent sex				4.62 (2)	0.099
Female	35 (26.12)	62 (46.27)	37 (27.61)		
Male	20 (17.09)	52 (44.44)	45 (38.46)		
Parent sex				4.07 (2)	0.130
Female	42 (25.77)	71 (43.56)	50 (30.67)		
Male	13 (14.77)	43 (48.86)	32 (36.36)		
Parent age				6.75 (2)	0.034
<35	25 (29.07)	41 (47.67)	20 (23.26)		
≥35	30 (18.18)	73 (44.24)	62 (37.58)		
Parent education				5.33 (2)	0.069
HS or less	22 (28.57)	27 (35.06)	28 (36.36)		
More than HS	33 (18.97)	87 (50.00)	54 (31.03)		
Household income				3.46 (2)	0.177
Less than $45,000	27 (27.27)	41 (41.41)	31 (31.31)		
$45,000 or more	26 (17.45)	72 (48.32)	51 (34.23)		
Marital status				6.66 (2)	0.036
Married	33 (20.00)	69 (41.82)	63 (38.18)		
Not married	22 (25.58)	45 (52.33)	19 (22.09)		
Parents’ weight status				1.66 (4)	0.798
Normal	18 (19.78)	39 (42.86)	34 (37.36)		
Overweight	13 (18.84)	33 (47.83)	23 (33.33)		
Obese	14 (25.45)	25 (45.45)	16 (29.09)		
Adolescents’ weight status				1.81 (4)	0.770
Normal	24 (19.35)	59 (47.58)	41 (33.06)		
Overweight	11 (25.00)	17 (38.64)	16 (36.36)		
Obese	12 (24.00)	24 (48.00)	14 (28.00)		

Note: Chi-square test.

**Table 3:** Weekly food group servings by family meal frequency among Hispanic adolescents.

	2 or Fewer Median (IQR)	3–6 Times Median (IQR)	7 or More Median (IQR)	χ^2^ (*df*)	*p*-Value
Dairy	11.50 (6.50–21.0)	14.25 (8.5–23)	11.5 (6.5–23)	2.00 (2)	0.367
Fruit	13.5 (5.5–23.3)	16.25 (7.5–25.5)	13.75 (8–28)	1.90 (2)	0.386
Grains	12.0 (6.5–22.5)	20.25 (12.0–29.0)	16.75 (9–32)	7.77 (2)	0.020
Protein	18.25 (10.5–29.75)	21.5 (11.5–34)	17.62 (12.5–27.12)	3.17 (2)	0.205
Vegetables	12.5 (4.5–25)	14.75 (7.5–30.0)	13.50 (5–27.5)	0.90 (2)	0.637

Note: Kruskal–Wallis test.

**Table 4:** General parenting styles and food parenting practices by family meal frequency.

	2 or Fewer Median (IQR)	3–6 Times Median (IQR)	7 or More Median (IQR)	χ^2^ (*df*)	*p*-Value
General Parenting Styles					
Responsiveness	4.20 (3.20–4.80)	4.20 (3.80–5.0)	4.8 (4.20–5.0)	15.35 (2)	<0.001
Involvement	4.40 (3.20–5.0)	4.60 (4.0–5.0)	5.0 (4.40–5.0)	12.59 (2)	0.002
Inconsistent Discipline	3.60 (2.80–4.80)	3.80 (3.0–4.40)	3.60 (2.60–4.60)	1.91 (2)	0.384
Expectation	4.0 (3.20–5.0)	4.40 (3.80–5.00)	4.80 (4.0–5.0)	8.30 (2)	0.016
Psychological Control	3.20 (2.40–4.40)	3.40 (2.60–4.20)	3.20 (2.0–4.40)	0.72 (2)	0.697
Authoritarian	3.80 (3.0–4.6)	3.70 (3.20–4.20)	3.60 (3.0–4.20)	1.29 (2)	0.525
Excessive Involvement	3.69 (3.0–4.40)	3.60 (3.0–4.20)	3.80 (3.20–4.20)	1.66 (2)	0.435
Food Parenting Practices					
Restriction	3.40 (3.0–4.40)	3.60 (3.0–4.0)	3.80 (3.0–4.40)	3.06 (2)	0.217
Modeling	3.33 (2.93–3.73)	3.40 (3.0–3.93)	3.67 (3.0–4.27)	4.40 (2)	0.110
Monitoring	3.0 (2.14–3.86)	3.28 (2.71–3.86)	3.71 (3.0–4.43)	12.83 (2)	0.002
Encouragement	3.17 (2.67–4.0)	3.33 (2.83–4.0)	4.08 (3.33–4.50)	22.91 (2)	<0.001

Note: Kruskal–Wallis test.

**Table 5:** Adjusted associations between family meal frequency and general parenting styles and food parenting practices.

	3–6 Times/Week vs. ≤2 Times/Week	≥7 Times/Week vs. ≤2 Times/Week	Overall *p*
General Parenting Styles			
Responsiveness	0.11 (−0.16, 0.38), *p* = 0.418	0.37 (0.10, 0.65), *p* = 0.008	0.009
Involvement	0.20 (−0.08, 0.48), *p* = 0.154	0.44 (0.15, 0.73), *p* = 0.003	0.006
Inconsistent Discipline	−0.09 (−0.44, 0.25), *p* = 0.594	−0.36 (−0.77, 0.04), *p* = 0.079	0.168
Expectation	0.23 (−0.06, 0.51), *p* = 0.125	0.38 (0.07, 0.68), *p* = 0.015	0.047
Psychological Control	−0.13 (−0.54, 0.28), *p* = 0.531	−0.27 (−0.73, 0.18), *p* = 0.243	0.495
Authoritarian	−0.22 (−0.55, 0.10), *p* = 0.175	−0.24 (−0.59, 0.10), *p* = 0.162	0.323
Excessive Involvement	−0.10 (−0.41, 0.20), *p* = 0.505	0.04 (−0.29, 0.36), *p* = 0.828	0.520
Food Parenting Practices			
Restriction	0.03 (−0.28, 0.33), *p* = 0.868	0.17 (−0.16, 0.50), *p* = 0.307	0.449
Modeling	0.05 (−0.21, 0.31), *p* = 0.730	0.20 (−0.10, 0.50), *p* = 0.191	0.304
Monitoring	0.20 (−0.14, 0.54), *p* = 0.250	0.49 (0.10, 0.87), *p* = 0.013	0.037
Encouragement	0.04 (−0.26, 0.33), *p* = 0.815	0.55 (0.22, 0.88), *p* = 0.001	<0.001

Note: Values are β (95% CI) and *p*-value from multivariable linear regression models (HC3-robust standard errors) with family meal frequency ≤2 times/week as the reference category, adjusted for adolescent age and sex, parent age, sex, education, income, and marital status. Overall *p* is from a 2-degree-of-freedom Wald test. N = 248 (complete-case analysis).

**Table 6:** Adjusted associations between family meal frequency and adolescent weekly food group intake.

	3–6 Times/Week vs. ≤2 Times/Week	≥7 Times/Week vs. ≤2 Times/Week	Overall *p*
Dairy	1.94 (−2.69, 6.57), *p* = 0.412	4.03 (−1.97, 10.03), *p* = 0.188	0.402
Fruit	2.20 (−2.46, 6.85), *p* = 0.355	6.59 (0.78, 12.41), *p* = 0.026	0.080
Grains	3.71 (−2.06, 9.47), *p* = 0.207	8.19 (0.65, 15.72), *p* = 0.033	0.101
Protein Foods	2.93 (−2.37, 8.23), *p* = 0.278	4.53 (−2.36, 11.42), *p* = 0.197	0.370
Vegetables	0.82 (−4.49, 6.14), *p* = 0.762	4.26 (−2.33, 10.85), *p* = 0.205	0.410

Note: Values are β (95% CI) and *p*-value from multivariable linear regression models (HC3-robust standard errors) with family meal frequency ≤2 times/week as the reference category, adjusted for adolescent age and sex, parent age, sex, education, income, and marital status. Overall *p* is from a 2-degree-of-freedom Wald test. β values represent weekly servings. N = 248 (complete-case analysis).

## Data Availability

The data presented in this study are available on request from the corresponding author. The data are not publicly available due to privacy and confidentiality protections for adolescent and parent participants.
